# Rapidly Acquired Resistance to EGFR Tyrosine Kinase Inhibitors in NSCLC Cell Lines through De-Repression of FGFR2 and FGFR3 Expression

**DOI:** 10.1371/journal.pone.0014117

**Published:** 2010-11-29

**Authors:** Kathryn E. Ware, Marianne E. Marshall, Lydia R. Heasley, Lindsay Marek, Trista K. Hinz, Paula Hercule, Barbara A. Helfrich, Robert C. Doebele, Lynn E. Heasley

**Affiliations:** 1 Department of Craniofacial Biology, University of Colorado at Denver, Aurora, Colorado, United States of America; 2 Department of Medicine, University of Colorado at Denver, Aurora, Colorado, United States of America; National Cancer Institute, United States of America

## Abstract

Despite initial and sometimes dramatic responses of specific NSCLC tumors to EGFR TKIs, nearly all will develop resistance and relapse. Gene expression analysis of NSCLC cell lines treated with the EGFR TKI, gefitinib, revealed increased levels of FGFR2 and FGFR3 mRNA. Analysis of gefitinib action on a larger panel of NSCLC cell lines verified that FGFR2 and FGFR3 expression is increased at the mRNA and protein level in NSCLC cell lines in which the EGFR is dominant for growth signaling, but not in cell lines where EGFR signaling is absent. A luciferase reporter containing 2.5 kilobases of *fgfr2* 5′ flanking sequence was activated after gefitinib treatment, indicating transcriptional regulation as a contributing mechanism controlling increased FGFR2 expression. Induction of FGFR2 and FGFR3 protein as well as *fgfr2*-luc activity was also observed with Erbitux, an EGFR-specific monoclonal antibody. Moreover, inhibitors of c-Src and MEK stimulated *fgfr2*-luc activity to a similar degree as gefitinib, suggesting that these pathways may mediate EGFR-dependent repression of FGFR2 and FGFR3. Importantly, our studies demonstrate that EGFR TKI-induced FGFR2 and FGFR3 are capable of mediating FGF2 and FGF7 stimulated ERK activation as well as FGF-stimulated transformed growth in the setting of EGFR TKIs. In conclusion, this study highlights EGFR TKI-induced FGFR2 and FGFR3 signaling as a novel and rapid mechanism of acquired resistance to EGFR TKIs and suggests that treatment of NSCLC patients with combinations of EGFR and FGFR specific TKIs may be a strategy to enhance efficacy of single EGFR inhibitors.

## Introduction

A general goal of molecular studies in cancer is to dissect the dominant oncogenic pathways and highlight specific components of these pathways as therapeutic targets. In this manner, the EGFR has emerged over the past years as an important target that likely plays key roles in non-small cell lung cancer (NSCLC) [1], [2]. The small molecule tyrosine kinase inhibitors (TKIs), gefitinib and erlotinib, were developed and deployed as experimental therapeutics in NSCLC [3]. While the spectrum of NSCLC patients that exhibit objective responses to gefitinib or erlotinib is disappointingly narrow [4], the positive activity on a defined subpopulation of NSCLC patients in which EGFR is a dominant oncogene is reason for optimism for continued development of novel targeted therapeutics to other oncogenes in lung cancer.

Among the NSCLC patients who initially respond to EGFR TKIs, all will eventually relapse (reviewed [5]). In fact, acquired resistance to single targeted molecular therapies is a general problem in cancer treatment. Chronic myelogenous leukemia (CML) patients treated with imatinib, a BCR-Abl inhibitor, can undergo relapse due to the acquisition of a secondary mutation within the Abl coding sequence [6], [7] that renders imatinib ineffective. Similarly, EGFR TKI responsive patients acquire secondary mutations, T790M in the ATP binding cleft, which increases EGFR affinity for ATP (reviewed in [8]). Aside from acquisition of secondary mutations, alternative receptor tyrosine kinase signaling can lead to acquired resistance to EGFR TKIs. For example, c-Met amplifications following EGFR TKI treatment contribute to acquired resistance to EGFR TKIs [9]. IGF-IR has also been reported to be hyperphosphorylated following EGFR TKI treatment [10]. Therefore, alternative signaling pathways mediating self-sufficiency in growth will need to be identified and targeted.

Clinical and biological evidence suggest that EGFR signaling is only one important signaling pathway in lung cancer. If self-sufficiency in growth is a hallmark of cancer, then additional receptor tyrosine kinases capable of signaling for growth, which render EGFR autocrine signaling redundant, can account for the reduced effectiveness of EGFR TKIs in lung cancer. Multiple studies support the hypothesis that EGFR independent receptor tyrosine kinase signaling pathways are active in EGFR TKI insensitive NSCLC [11], [12], [13]. In particular FGFR autocrine signaling has been implicated in NSCLC cell lines [11]. FGFs and their receptors (FGFRs) are involved in multiple cellular functions. During embryonic development FGFs play a role in morphogenesis through cell proliferation, differentiation, and cell migration. In adults, FGFs are involved in wound healing and tissue repair as well as regulating the nervous system. Unfortunately, they also contribute to tumor angiogenesis [14], [15]. Numerous *in vitro* studies reveal frequent co-expression of specific FGFs as well as FGFR1 and FGFR2 [11], [13], [16], [17], [18], [19]. Primary NSCLC specimens also show co-expression of FGF2, FGFR1, and FGFR2 [20]. Importantly, inhibition of FGFR signaling via dominant-negative FGFR1 [18], FGF2 neutralizing antibodies [19], FGFR TKI [11] or anti-sense RNA [11], [19] approaches blocked proliferation of tumor growth in NSCLC. These studies suggest FGF-FGFR co-expression can function as an autocrine growth pathway, particularly in NSCLC cells lines intrinsically resistant to EGFR TKIs [11]. In this study, we present evidence for a novel role of FGFR2 and FGFR3 in acquired resistance to EGFR TKIs in NSCLC cells.

## Results

### FGFR2 and FGFR3 expression is induced after EGFR inhibition

**Figure 1:**
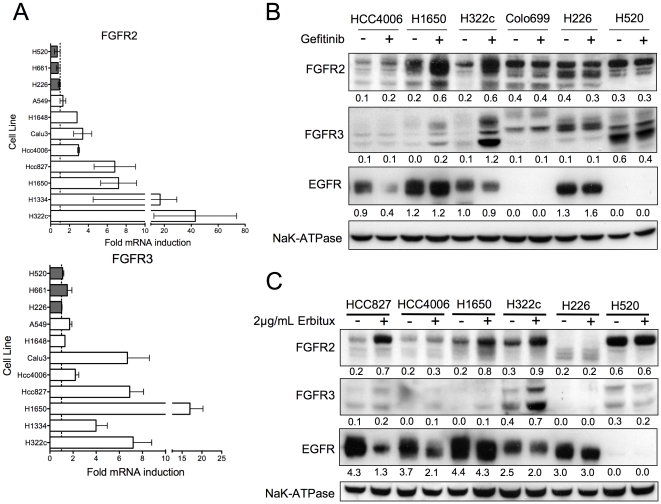
Induction of FGFR2 and FGFR3 mRNA and protein in EGFR inhibitor treated NSCLC cells. A. Quantitative RT-PCR assay for FGFR2 and FGFR3 mRNAs. Total RNA was purified from the indicated cell lines following a 3-day treatment with 1 µM gefitinib and submitted to quantitative RT-PCR analysis of FGFR2, FGFR3 and GAPDH. (Cell lines with EGFR autocrine signaling or gain-of-function EGFR mutations: open bars; cell lines lacking EGFR signaling: grey bars) The data are presented as fold expression over DMSO treated cells following normalization for GAPDH mRNA. B–C. Cell lysates from the indicated NSCLC cell lines treated 3 days with or without 1 µM gefitinib or 2 µg/mL Erbitux were immunoblotted for FGFR2, FGFR3, EGFR and the α-subunit of the NaK-ATPase as a loading control. Densitometry of FGFR2, FGFR3 and EGFR expression was normalized relative to NaK-ATPase expression and is indicated under each immunoblot.

Total RNA from H322c NSCLC cells treated 4 days with DMSO (0.1%) as a control or with the EGFR TKI, gefitinib, was purified and used to probe Affymetrix human U133 plus 2.0 arrays. Gene expression changes detected by microarray analysis revealed induction of FGFR2 and FGFR3 but not FGFR1, FGFR4, or FGFR ligands in gefitinib treated cells (Table S1). Other tyrosine kinases, such as Met and IGF1R, which are reported to be important for acquired resistance to EGFR inhibitors [9], [10], were not induced over control treatment. Quantitative RT-PCR analysis of 9 NSCLC cell lines previously characterized for sensitivity to the EGFR inhibitor gefitinib [21] and the FGFR inhibitor RO4383596 [11] confirmed the induction of FGFR2 and FGFR3 expression changes in a larger panel of NSCLC cells. Interestingly, FGFR2 and FGFR3 expression was induced in all NSCLC cells that have been shown to be gefitinib sensitive (H322c, HCC827, HCC4006) and correlated with cells that co-express EGFR and EGF ligands (H322c, H1334, Calu3) or bear gain-of-function EGFR (HCC827, HCC4006, H1650) (Figure 1A). NSCLC cells that do not express EGFR (H661, H520) or are resistant to gefitinib (H226) [11] did not exhibit FGFR2 and FGFR3 mRNA induction in response to gefitinib (Figure 1A). This indicates that FGFR induction in response to gefitinib is not due to off-target effects of the drug, but is related to targeted effects on functional EGFR signaling. FGFR2 and FGFR3 protein levels as assessed by immunoblot analysis coincided with FGFR2 and FGFR3 mRNA measured by quantitative RT-PCR. As shown in Figure 1B, gefitinib induces FGFR2 and FGFR3 at the protein level in cells co-expressing EGFR and EGF ligands or gain-of-function EGFR. NSCLC cells which do not express EGFR (Colo699, H520) or respond to gefitinib (H226), do not undergo induction of FGFR2 or FGFR3 (Figure 1B). Consistent with a specific effect of gefitinib on the EGFR, Erbitux, a monoclonal antibody specifically targeting the EGFR, similarly induces FGFR2 and FGFR3 expression in the same NSCLC cell lines that are responsive to gefitinib (Figure 1C). Finally, partial knockdown of the EGFR with siRNA leads to increased FGFR2 expression (Figure S1). Notably, gefitinib treatment also induces FGFR2 protein in MCF-7 cells, a breast cancer cell line, and 3 different head and neck cancer cell lines (UMSCC2, UMSCC8, and HN31, Figure S1). This suggests that the mechanism by which gefitinib induces FGFR2 and FGFR3 is likely to be operative in diverse epithelial-derived cancer cell lines. To further test if FGFR2 and FGFR3 are repressed downstream EGFR signaling, H226 cells, which express high levels of FGFR2 and FGFR3, were incubated with 10 ng/mL EGF for 36 hrs. As shown in Figure S1, EGFR activation inhibited FGFR2 and FGFR3 protein expression but not FGFR1 expression in H226 cells. Combined, these experiments suggest that FGFR2 and FGFR3 expression is repressed downstream of EGFR signaling such that EGFR TKI treatment allows for FGFR2 and FGFR3 expression.

### FGFR2 expression is regulated transcriptionally post gefitinib treatment

**Figure 2:**
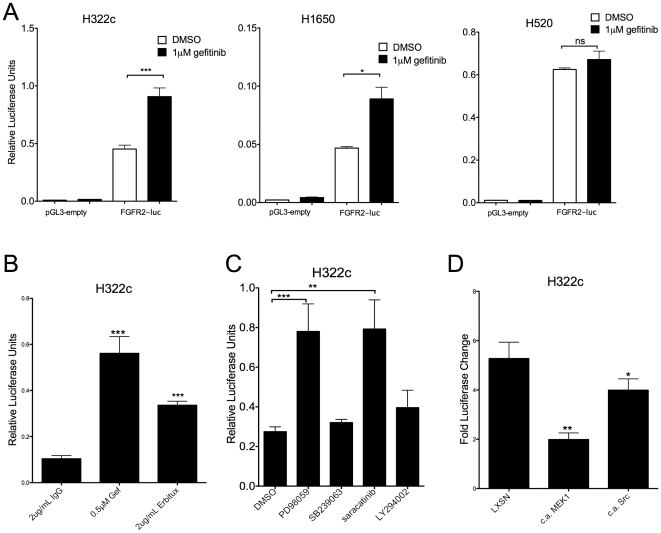
Transcriptional regulation of FGFR2 following EGFR inhibitor treatment. A. H322c, H1650 or H520 cells transfected with pGL3-basic empty control vector or FGFR2-luc reporter (see Material and Methods) and TK-renilla to estimate transfection efficiency were treated with 1 µM gefitinib for 48 hrs. Cells were then lysed and luciferase activity measured and normalized to renilla activity. The data are the mean and SEM of 3 independent experiments. B. H322c cells transfected as above were treated with gefitinib (0.5 µM) or 2 µg/mL Erbitux for 48 hrs. The data are the mean and SEM of 4 independent experiments. C. FGFR2-luc transfected H322c cells were treated with the indicated inhibitors (5 µM PD98059, 1 µM saracatinib, 5 µM SB239063, or 10 µM LY294002) for 48 hrs. Luciferase expression was then measured as described above. Data are the mean and SEM of 3 independent experiments. D. H322c cells co-transfected with FGFR2-luc and empty LXSN, constitutively active MEK1 or constitutively active c-Src were treated for 48 hrs with 0.5 µM gefitinib. Luciferase expression was measured as described above. Data are the mean and SEM of 4 independent experiments. Statistical analysis by two-tailed t-test revealed significant increases in luciferase activity where ns indicates not significant, * indicates p<0.05, ** indicates p<0.005 and *** indicates p<0.001.

To determine the kinetics of FGFR2 and FGFR3 induction, quantitative RT-PCR was used to measure FGFR2 and FGFR3 mRNA expression over 4 days. In H322c cells, FGFR2 mRNA is maximally induced within 24–48 hrs of gefitinib treatment while FGFR3 mRNA accumulates more slowly (Figure S2). Interestingly, FGFR2 and FGFR3 induction occurs quickly (1–2 days) relative to the previously reported Met gene amplification in response to gefitinib which required ∼6 months [9]. The rapid induction of FGFR2 and FGFR3 mRNA suggests that the *fgfr2* and *fgfr3* genes are not amplified but are being regulated at the transcriptional level. To test whether mRNA levels are regulated transcriptionally or post-transcriptionally, the 5′ flanking region of the *fgfr2* gene (accession number: NT_030059) containing the basal *fgfr2* promoter [22] was cloned from genomic DNA (see Materials and Methods) and ligated upstream of the luciferase gene in pGL3-basic. The *fgfr2*-luc reporter was then transiently transfected into H322c and H1650 cells, followed by treatment with or without 1 µM gefitinib for 48 hrs. FGFR2 promoter activity significantly increased after gefitinib treatment, whereas the empty vector showed no change in activity following gefitinib treatment (Figure 2A). Thus, increased FGFR2 mRNA is, in part, mediated by transcriptional induction of the *fgfr2* gene following gefitinib treatment. Consistent with the failure of gefitinib to induce FGFR2 and FGFR3 protein in NSCLC cell lines lacking EGFR (Figure 1B), gefitinib treatment had no effect on *fgfr2-*luc activity in the EGFR-null NSCLC cell line, H520 (Figure 2A). Likewise, the monoclonal antibody, Erbitux, significantly increased FGFR2 promoter activity (Figure 2B). Next, *fgfr2*-luc transfected cells were treated with inhibitors of downstream effectors of EGFR signaling, MEK (PD98059), PI3K (LY294002), c-Src (saracatinib) and p38 MAP kinase (SB239063), for 48 hrs. MEK inhibitor, PD98059, and c-Src inhibitor, saracatinib, showed a similar induction of FGFR2 transcription activity as gefitinib treatment (Figure 2C), indicating that one or both of these signaling pathways mediate transcriptional repression of *fgfr2* downstream of the EGFR. To eliminate off-target effects of PD98059 and saracatinib, constitutively active MEK1 and c-Src were co-transfected with the *fgfr2*-luc construct in H322c cells, followed by treatment with or without gefitinib for 48 hrs. Constitutively active MEK1 and c-Src significantly reduced *fgfr2*-luc activity in response to gefitinib (Figure 2D).

### FGFR induction leads to FGF stimulated signaling through the ERK pathway

**Figure 3:**
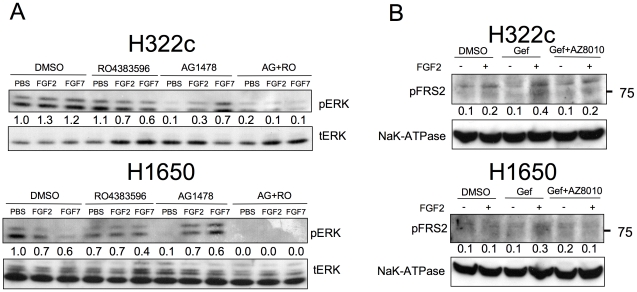
FGF-stimulated ERK and FRS2-α activation following EGFR TKI treatment. A. NSCLC cell lines were incubated with the EGFR inhibitor, AG1478 (0.1 µM) or DMSO, for 3 days in full media. Cells were switched to HITES for 2 hrs and subsequently incubated with the FGFR inhibitor, RO4383596 (1 µM) or DMSO for 1 hour followed by FGF2 or FGF7 stimulation at 10 ng/mL for 15 minutes. Extracts were prepared, resolved on SDS-PAGE and immunoblotted for phospho-ERK. The filters were subsequently stripped and reprobed for total ERK1 and ERK2 to verify equal loading. Densitometry of phospho-ERK2 relative to total ERK2 for the designated experiment is indicated. The mean and SEM of replicate experiments is indicated in the text of the Results section. B. NSCLC cell lines were incubated with the EGFR inhibitor, gefitinib (1 µM), gefitinib in combination with FGFR inhibitor AZ12908010, or DMSO for 3 days in full media. Cells were then stimulated with PBS or FGF2 at 10 ng/mL for 15 minutes. Extracts were prepared, resolved on SDS-PAGE and immunoblotted for phopho-FRS2. The filters were subsequently stripped and reprobed for NaK-ATPase α-subunit to verify equal loading. Densitometry of phospho-FRS2 relative to NaK-ATPase is indicated under each immunoblot.

The findings in Figures 1 and 2 demonstrate transcriptional induction of FGFR2 and FGFR3. To test whether induced FGFRs were capable of signaling, stimulation of the ERK pathway by exogenous FGFs was measured as a downstream target of FGFRs. H322c and H1650 cells were cultured for 3 days in the presence or absence of AG1478, an EGFR inhibitor, and then subsequently incubated with FGF2 or FGF7 for 15 minutes. Cell extracts were submitted to immunoblot analysis of phospho-ERK as a measure of receptor activation. While untreated H322c or H1650 cells show little or no increase in ERK activation when stimulated with FGF2 (1.5±0.2, 0.9±0.2 fold, respectively) or FGF7 (1.2±0.1, 0.6±0.2 fold, respectively) (Figure 3A lanes 2 and 3), cells cultured 72 hrs in the presence of AG1478 to induce FGFR2 and FGFR3 have significantly lower basal ERK activity (0.4±0.1, 0.2±0.1 fold, respectively) due to blockade of the EGFR pathway, (Figure 3A lane 7) but a marked increase in ERK phosphorylation in response to FGF2 (5.0±1.2, 10.7±4.2 fold, respectively) and FGF7 (6.4±1.0, 8.9±5.7 fold, respectively) (Figure 3A lanes 8 and 9). To define that ERK activation after AG1478 treatment is FGFR mediated, cells cultured for 3 days in the presence of AG1478 were pre-incubated with an FGFR TKI, RO4383596 [11], [23], 1 hr prior to FGF2 or FGF7 stimulation. This treatment with RO4383596 completely eliminated the FGF stimulated phospho-ERK response following AG1478 treatment (Figure 3A lanes 11 and 12), but has no effect on phospho-ERK when used alone (Figure 3A lanes 4, 5, and 6). Thus, an FGFR mediated activation of ERK is observed in both H322c and H1650 cells following a 3-day treatment with an EGFR specific TKI. Likewise, increased phosphorylation of FRS2 occurred in response to FGF2 stimulation after EGFR TKI treatment in H322c and H1650 cells (Figure 3B lane 4). This response could also be blocked with the FGFR-specific TKI (Figure S3), AZ12908010, demonstrating direct activation of the FGFR signaling pathway (Figure 3B).

### Exogenous FGF2 or FGF7 rescues growth of NSCLC cells following treatment with an EGFR specific TKI

**Figure 4:**
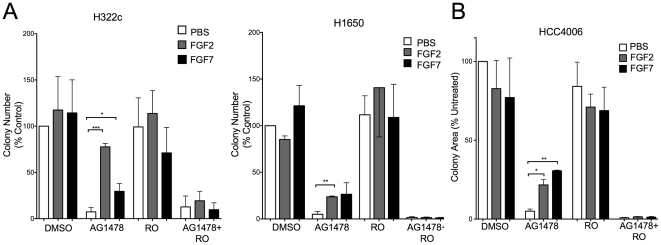
FGF2 and FGF7 rescue EGFR TKI dependent growth inhibition. A. H322c and H1650 cells were analyzed for anchorage-independent growth as described in the Materials and Methods. B. HCC4006 cells were seeded at 100 cells per 35 mm dish in full media containing the indicated treatments and cultured for 2 weeks. Colony area was then quantified and shown as a percentage of control. The data are the mean and SEM of 3 independent experiments. Statistical analysis by 2way ANOVA revealed significant increases in growth where * indicates p<0.05, ** indicates p<0.005 and *** indicates p<0.001.

The previous results show that NSCLC cells cultured with EGFR TKIs have increased FGFR2 and FGFR3 expression and increased ERK activation in response to FGF2 or FGF7. To directly test the role of FGFR2 and FGFR3 induction as a mechanism of resistance to EGFR TKI treatment, anchorage-independent growth of H322c and H1650 cells was measured. Cells were cultured in 0.35% agar overlaid with media containing combinations of AG1478, RO4383596, FGF2 or FGF7. Although neither FGF2 nor FGF7 alone or in combination with RO4383596 had a significant effect on colony number (Figure 4A), FGF2 or FGF7 significantly stimulated anchorage-independent growth of these cell lines with AG1478 treatment (Figure 4A). As predicted, this growth response was blocked by addition of RO4383596. In addition, HCC4006, which fails to robustly form colonies in soft agar, showed a similar response when stimulated with FGF2 or FGF7 in a clonogenic growth assay (Figure 4B, see Materials and Methods). Again, the ability of EGFR TKIs to induce FGFR2 and FGFR3 is not restricted to NSCLC cell lines. As shown in Figure S4, FGF2 stimulated clonogenic growth of HNSCC cell lines, UMSCC2 and HN31, upon treatment with gefitinib. Moreover, FGF2-stimulated growth of gefitinib treated cells was inhibited by an FGFR-specific TKI, AZ12908010 (Figure S4).

### Co-culture of H322c cells with human fibroblasts rescues EGFR TKI induced growth inhibition in an FGFR-dependent manner

**Figure 5:**
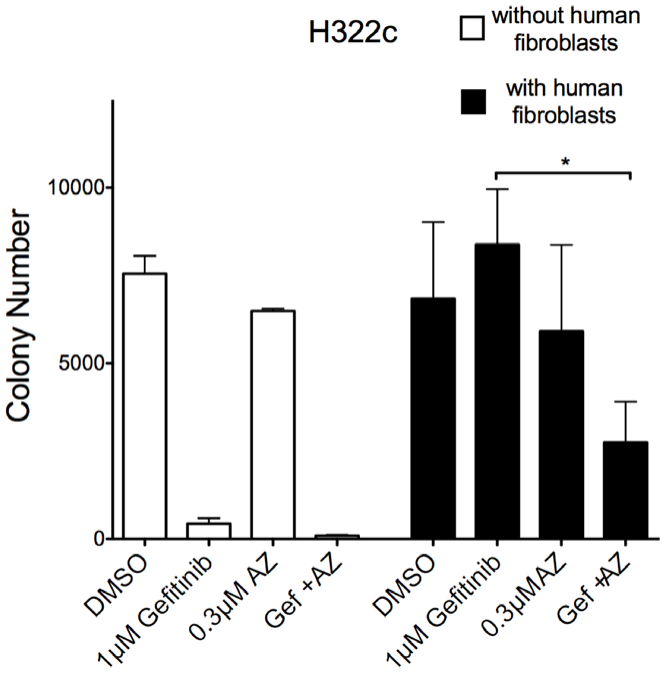
Co-culture with human fibroblasts prevents EGFR TKI dependent growth inhibition. A. H322c cells were analyzed for anchorage-independent growth in the presence or absence of HGF-1 fibroblasts as described in the Materials and Methods. The data are the mean and SEM of 3 independent experiments. Statistical analysis by 2way ANOVA revealed significant increases in growth where * indicates p<0.05.

Considering exogenous FGF2 and FGF7 can rescue anchorage independent growth, human fibroblasts were cultured as feeder layers with H322c cells in an anchorage-independent growth assay to test the role of paracrine-derived FGFs in FGFR mediated acquired resistance. As previously noted (Figure 4), H322c cells cultured in the absence of human fibroblasts were strongly growth inhibited by the EGFR TKI, gefitinib (Figure 5). By contrast, H322c cells cultured with a fibroblast feeder layer were not significantly inhibited by gefitinib (Figure 5). However, inclusion of the FGFR inhibitor, AZ12908010, with gefitinib significantly blocked anchorage-independent growth in the presence of a fibroblast feeder layer, although AZ12908010 has little effect on anchorage independent growth when used alone (Figure 5). Combined, our studies indicate that EGFR TKIs promote FGFR2 and FGFR3 as an alternate signaling pathway capable of communicating with the surrounding environment via secreted FGFs.

## Discussion

The six hallmarks of tumorigenesis outlined by Hanahan and Weinberg have been widely agreed upon and supported by the literature. However, therapies targeting signaling pathways critical to the manifestation of these hallmarks have shown both successes and failures. Even in successful cases such as imatinib targeting Bcr-Abl, secondary mutations arise contributing to acquired resistance in CML patients [6], [7]. Therefore, as methods of targeting oncogenic pathways in cancer cells advance, so will the necessity for targeting mechanisms of acquired drug resistance. Besides the accumulation of secondary mutations in targeted proteins, a simple explanation for acquired resistance is the selection for and emergence of alternative RTK systems. In NSCLC cell lines and tumors, an independent phosphoproteomic approach confirms the extensive array of RTKs that are expressed and active [24]. Thus, there are numerous RTK candidates that may function as alternatives to EGFR in signal transduction of growth and transformation in NSCLC. In fact, the notion of a receptor tyrosine kinase coactivation network has been posited as an alternative to single RTKs working in isolation [25]. In NSCLC, a role for c-Met and IGF-1R has been most extensively explored [5], [12], [26]. In response to gefitinib treatment, amplification of chromosomal region 7q31.1–33.3 containing the c*-Met* gene has been observed, allowing c-Met activation of the PI3K/AKT pathway in an ErbB3-dependent manner, but independent of either EGFR or ErbB2 activation [9]. Furthermore, it was shown that acquired resistance to gefitinib was associated with hyperphosphorylation of the IGF-1R receptor and constitutive association with PI3K [10]. Our data highlights the FGFR pathway as yet another RTK system that may contribute to acquired resistance to EGFR TKIs. Therefore, acquisition of resistance may take months as in c-Met gene amplification [9], weeks as in IGF-1R activation [10], [27], or days as in the transcriptional regulation of FGFR2 and FGFR3 shown in this study. In this regard, Sharma et al. [27] have shown that cancer cell lines undergo a rapid and reversible epigenetic response to diverse growth inhibitors, including gefitinib, that results in drug resistance.

The biological significance of FGFR2 and FGFR3 being repressed in NSCLC cells in which the EGFR pathway is activated is a question not completely addressed herein. Interestingly, FGFR2 and FGFR3 mRNA expression is increased in human bronchial epithelial cells (HBEC) grown at the air water interface to promote differentiation into a pseudostratified epithelium consisting of mucous secreting and ciliated cells (Figure S5) [28]. This finding suggests that high expression of FGFR2/FGFR3 and downstream signaling may be associated with a more differentiated epithelial state. Preliminary data from our lab supports an ability of EGFR TKIs to promote induction of epithelial differentiation. Similar increases in specific measures of epithelial differentiation in response to EGFR TKIs have also been observed in squamous cancer cell lines [29]. In normal lung, FGFR2 functions in epithelial cells and alveolar type II pneumocytes to promote proliferation and differentiation, thereby contributing to lung morphogenesis and repair [30], [31]. However, FGFR2 signaling in the tumor setting, where FGF2 and FGF7 are likely available from the surrounding microenvironment [30], [32], may establish a paracrine pathway leading to continued cancer cell survival, growth and maintenance of transformation in the presence of EGFR targeted therapies.

A clear theme arising from clinical trials with single targeted therapeutic agents is the emergence of acquired resistance mechanisms, both delayed and rapid [26], [27]. This likely reflects a Darwinian adaptive process at the cellular level, leading to accumulation of second-site point mutations (reviewed in [8]), gene amplification, increased activation of distinct receptor tyrosine kinases [9], [10] or transcriptional induction of alternative growth factor receptor pathways as detailed herein. This presents the obvious need for strategies in which an inhibitor of the primary molecular target and one or more inhibitors of dominant resistance mechanisms are deployed simultaneously or consecutively to enhance initial tumor cell killing or prolong an anti-tumor response. Thus, one scenario would entail the use of an FGFR inhibitor in combination with EGFR TKIs to prevent rapid surmounting of growth inhibition mediated by FGFR2 and/or FGFR3 induction. Obviously, a modern targeted therapy that predicts the correct combination of TKIs and other inhibitors becomes very complex. Clearly, molecular based medicine for treatment of cancer patients must advance whereby serial assessment of tumor markers is performed to properly choose initial drug treatment regimens as well as second and third stage treatments to combat evolving resistance mechanisms.

## Materials and Methods

### Cell Culture

All NSCLC cell lines except Colo699, H661, and H226 were previously described by Coldren et al. [21]. The remaining lines were obtained from the University of Colorado Cancer Center tissue culture core. HNSCC cancer cells lines (UMSCC2, UMSCC8, HN31) were previously described by Frederick et al. [33]. The NSCLC and HNSCC cell lines employed in this study were submitted to fingerprint analysis by the University of Colorado Cancer Center to verify their authenticity. All cell lines were routinely cultured in RPMI-1640 or DMEM (UMSCC8, UMSCC2, HN31) growth medium supplemented with 10% fetal bovine serum (Sigma, St. Louis, MO) at 37°C in a humidified 5% CO2 incubator. Where indicated, the cells were switched to HITES medium (RPMI-1640 containing 10 nM hydrocortisone, 5 µg/ml insulin, 10 µg/ml transferrin, 10 nM estradiol, 30 nM Na_3_SeO_3_ and 1% bovine serum albumin) to limit mitogenic inputs from serum components. HGF-1 fibroblasts were obtained from ATCC (Manassas, VA).

### Microarray Data

Total RNA prepared from control and gefitinib-treated H322c cells was used to probe Affymetrix U133 Plus 2.0 arrays within the University of Colorado Cancer Center Gene Expression Core. All data are MIAME compliant and the raw data has been deposited in a MIAME compliant database, GEO. (Accession #: pending)

### Quantitative Real-Time PCR (RT-PCR) Assay

Total RNA (5 µg) was reverse transcribed in a volume of 20 µl using random hexamers and MMLV reverse transcriptase (Invitrogen, Carlsbad, CA). Aliquots (5 µl) of 25-fold diluted reverse transcription reactions were subjected to PCR in 25 µl reactions with SYBR® green Jumpstart Taq Readymix (Sigma, St. Louis, MO) and the primers previously described for FGFR2 [11] or forward primer 5′-CCA TCG GCA TTG ACA AGG AC-3′ and reverse primer 5′-GCA TCG TCT TTC AGC ATC TTC AC-3′ for FGFR3 using a My iQ real time-PCR detection system (BioRad, Hercules, CA). GAPDH mRNA levels were measured by quantitative RT-PCR in replicate samples as a housekeeping gene for normalization of the different mRNA expression and the data are presented as “Relative Expression”.

### Clonogenic and Anchorage-Independent Growth Assays

To measure the effect of EGFR TKIs on clonogenic growth, cells were plated in 6-well plates at 100 cells/well and cultured in full growth medium with or without 0.1 µM AG1478 (Calbiochem San Diego, CA), 1 µM RO4383596 (Hoffmann-La Roche), 10 ng/mL FGF2 or FGF7 (PeproTech, Rocky Hill, NJ) for 2 weeks. Colonies were rinsed with PBS, stained with 200 ml of 6% (vol/vol) glutaraldehyde, 0.5% (wt/vol) crystal violet in H_2_O for 30 min. at room temperature and rinsed extensively in H_2_O [34]. Following digital photography, the total colony area was quantified using the MetaMorph imaging software program (Molecular Devices, Downingtown, PA).

For measurement of anchorage-independent cell growth, 40,000 cells (H322c) or 20,000 cells (H1650) were suspended in 1.5 mL RPMI 1640 containing 10% fetal bovine serum and 0.35% DifcoTM agar noble (Becton, Dickinson and Co., Sparks, MD) and overlaid on base layers containing 1.5 mL RPMI 1640 containing 10% fetal bovine serum and 0.5% agar noble in 6-well plates. The wells were covered with 2 mL growth medium containing drugs and growth factors (0.1 µMAG1478, 1 µMRO4383596, 10 ng/mL FGF2 or FGF7). This media was replaced with fresh media containing drugs and growth factors every 7 days. In experiments with human fibroblasts, HGF-1, co-culture cells were plated 1 day prior to being overlaid with agar as described above. The plates were incubated in a 37°C CO2 incubator for 21 days after which viable colonies were stained for 24 hrs with 200 mL of 1 mg/mL nitroblue tetrazolium. Following digital photography, the colony number was quantified using the MetaMorph imaging software program.

### Immunoblot Analyses

For analysis of phospho-ERK, cells were seeded in 6-well dishes to allow cell attachment. After 24 hrs, cells were treated with DMSO or 0.1 µM AG1478 for 72 hrs after which media was switched to HITES plus DMSO or AG1478 for another 2 hrs. Subsequently, the cells were then treated with 1 µM RO4383596 or DMSO for 1 hr, after which cells were stimulated with 10 ng/mL of FGF2, FGF7 or PBS for 15 min. For phospho-FRS2 analysis, cells were seeded in 10 cm dishes and allowed to attach. After 24 hrs, cells were treated with DMSO, 1 µM gefitinib (AstraZeneca, UK), or 1 µM gefitinib in combination with 0.3 µM AZ12908010 (AstraZeneca, UK), for 72 hrs after which media was refreshed with drugs 1 hr prior to stimulation with 10 ng/mL FGF2 or PBS for 15 min. Growth factor and/or drug-treated NSCLC cells were collected in 1 mL phosphate-buffered saline, centrifuged at 1,000× g for 5 min, lysed in MAP kinase lysis buffer (MKLB; 0.5% Triton X-100, 50 mM β-glycerophosphate (pH 7.2), 0.1 mM Na_3_VO_4_, 2 mM MgCl_2_, 1 mM EGTA, 1 mM DTT, 0.3 M NaCl, 2 µg/ml leupeptin and 4 µg/ml aprotinin) and centrifuged (5 min at 13,000 RPM). The particulate fractions were discarded and 10 µg (phospho-ERK) or 200 µg (phopho-FRS2) of the soluble extracts were mixed with SDS sample buffer and submitted to SDS-PAGE. Following electrophoretic transfer onto nitrocellulose, the filters were blocked in 3% bovine serum albumin (Cohn Fraction V, ICN Biomedicals, Inc., Aurora, OH) in Tris-buffered saline with 0.1% Tween 20 (TTBS) and then incubated with anti-phospho-ERK or phospho-FRS2-α (Cell Signaling Technology, Inc, Danvers, MA, #4377 and #3864) for 16 hours at 4°C. The filters were washed thoroughly in TTBS, then incubated with alkaline phosphatase coupled goat anti-rabbit antibodies and developed with LumiPhos reagent (Pierce, Rockford, IL) according to the manufacturer's instructions. The filters were subsequently stripped and reprobed for total ERK1 and ERK2 or NaK-ATPase using a mixture anti-ERK1 (sc-93) and ERK2 (sc-154) or NaK-ATPase α-subunit (sc-21712) antibodies (Santa Cruz Biotechnology, Inc., Santa Cruz, CA). For immunoblot analysis of FGFR1, FGFR2, FGFR3, EGFR and the α-subunit of NaK-ATPase, NSCLC cells were collected in phosphate-buffered saline, centrifuged (5 min, 1000× g) and suspended in MKLB after treatment with 1 µM gefitinib or 2 µg/mL Erbitux (Bristol-Myers Squibb, New York, NY). Aliquots of the cell lysate preparations containing 75 µg of protein were submitted to SDS-PAGE and immunoblotted for FGFR1 (sc-121), FGFR2 (sc-122), FGFR3 (sc-13121) and NaK-ATPase α-subunit (sc-21712) with antibodies from Santa Cruz Biotechnology (Santa Cruz, CA). EGFR was detected with a rabbit polyclonal antibody (#2232) from Cell Signaling Technology, Inc.

### Construction of FGFR2 Promoter Luciferase Plasmid and Luciferase Reporter Assays

Human genomic DNA (50 ng) was submitted to PCR using Phusion polymerase, forward primer 5′-GCCATTGACGAAAGGGTTC-3′ and reverse primer 5′-TGCCTCCACCAAACTTTGCTC-3′ that anneal at −2165 and +267 relative to the published transcription start site of human *fgfr2* [22]. The first 10 cycles were annealed at 67°C, 10°C above the T_m_, followed by 20 cycles with an annealing temperature at 57°C. Purified PCR products were then cloned into pCR-Blunt using the Zero Blunt cloning kit from Invitrogen (Carlsbad, CA) and submitted to DNA sequencing. The FGFR2 promoter region (−2165 to +267) was shuttled into pGL3-basic (Promega, Madison, WI) using KpnI and SmaI sites. The ligation junctions in the FGFR2 promoter luciferase vector were verified by DNA sequencing. Cell lines were transfected in 6-well plates with 0.5 µg of pGL3-basic or pGL3 *fgfr2* (−2165 to +267) alone or in combination with LXSN-C.A. MEK1 [35] or C.A c-Src (Millipore, Billerica, MA) and 0.25 µg of TK-Renilla using Effectene transfection reagent (Qiagen, Germantown, MD) according to manufacture's protocol. Cells were then treated with inhibitor (1 µM gefitinib, 2 µg/mL Erbitux, 5 µM PD98059, 10 µM LY294002, 1 µM saracatinib or 5 µM SB239063) for 48 hrs at which time cells were lysed and assayed for luciferase activity with the dual luciferase reporter assay system (Promega) according to the manufacture's protocol.

## Supporting Information

Figure S1 FGFR2 protein is regulated downstream EGFR signaling in cancer cell lines of epithelial origin. A. Cell lysates from the indicated HNSCC (UMSCC2, UMSCC8, HN31) and breast cancer (MCF-7) cell lines that had been treated with or without 1μM gefitinib (72hrs) were immunoblotted for FGFR2 and the α-subunit of the NaK-ATPase as a loading control. B. H322c cells transfected with 3 independent EGFR siRNA or scramble control (Sigma, St. Louis, MO) were cultured 72 hrs and immunoblotted for the EGFR, FGFR2 and NaK-ATPase. C. H226 cells were cultured with PBS or EGF (10 ng/ml) for 72 hrs. Cell lysates were immunoblotted for FGFR1, FGFR2, FGFR3, EGFR and the α-subunit of the NaK-ATPase.
(1.42 MB TIF)

Figure S2 Rapid induction of FGFR2 and FGFR3 mRNA. Quantitative RT-PCR assay for FGFR2 and FGFR3 mRNAs after treatment with 1μM gefitinib for varying amounts of time was performed on total RNA from H322c cells and normalized for GAPDH mRNA levels. Data are shown as fold expression over DMSO treated cells at the indicated times. The results are a representative of 3 independent experiments.
(0.18 MB TIF)

Figure S3 AZ12908010 is a specific inhibitor of FGFR receptors. Human gingival fibroblasts (HGF-1) purchased from ATCC were treated for 2 hrs with or without 100 nM AZ12908010 and then for another 15 minutes with or without FGF2 (10 ng/mL), EGF (10 ng/mL), PDGF-BB (20 ng/mL) or IGF-1 (10 ng/mL) as indicated. Cell extracts were prepared and submitted to SDS-PAGE and immunoblotted for phospho-ERK. The filters were subsequently stripped and reprobed for total ERK1 and ERK2 to verify equal loading. Only FGF2-stimulated phospho-ERK was inhibited by AZ12908010.
(0.56 MB TIF)

Figure S4 FGF2 rescues EGFR TKI dependent growth inhibition in HNSCC cells. UMSCC8 and HN31 head and neck squamous cell carcinoma lines were submitted to the clonogenic growth assay in the presence and absence of gefitinib and/or AZ12908010, an FGFR specific TKI (see Supplementary Figure S3). Colonies were stained and quantified as described in Materials and Methods.
(1.26 MB TIF)

Figure S5 FGFR2 and FGFR3 mRNA are induced during human bronchial epithelial cell differentiation at the air-water interface. GEO Data Set GSE5264 containing Affymetrix Human Genome U133 Plus 2.0 arrays of human bronchial epithelial cells grown over a 28 day period at the air-water interface (28) were queried for expression of EGFR, FGFR2 and FGFR3 using the Affymetrix IDs EGFR (201983_s_at), FGFR2 (203638_s_at), and FGFR3 (204379_s_at). Following normalization for GAPDH expression, the data were plotted to show the relative mRNA expression of these genes over time at the air-liquid interface. The data points reflect the mean and SEM of the three independent experiments performed.
(0.42 MB TIF)

Table S1 Gene expression changes in response to gefitinib treatment in H322c cells. Total RNA from H332c cells treated for 4 days with DMSO or 1 μM gefitinib was submitted to Affymetrix human U133 plus 2.0 arrays. Expression levels of selected tyrosine kinases and ligands are listed below. ("A" indicates absent and "P" present as assessed by the Affymetrix software program).
(0.37 MB DOC)
