# Supplementary figures and images for: Rapidly Acquired Resistance to EGFR Tyrosine Kinase Inhibitors in NSCLC Cell Lines through De-Repression of FGFR2 and FGFR3 Expression
Source: PLoS One. 2010 Nov 29;5(11):e14117. doi: 10.1371/journal.pone.0014117 (PMC2994708; doi:10.1371/journal.pone.0014117)

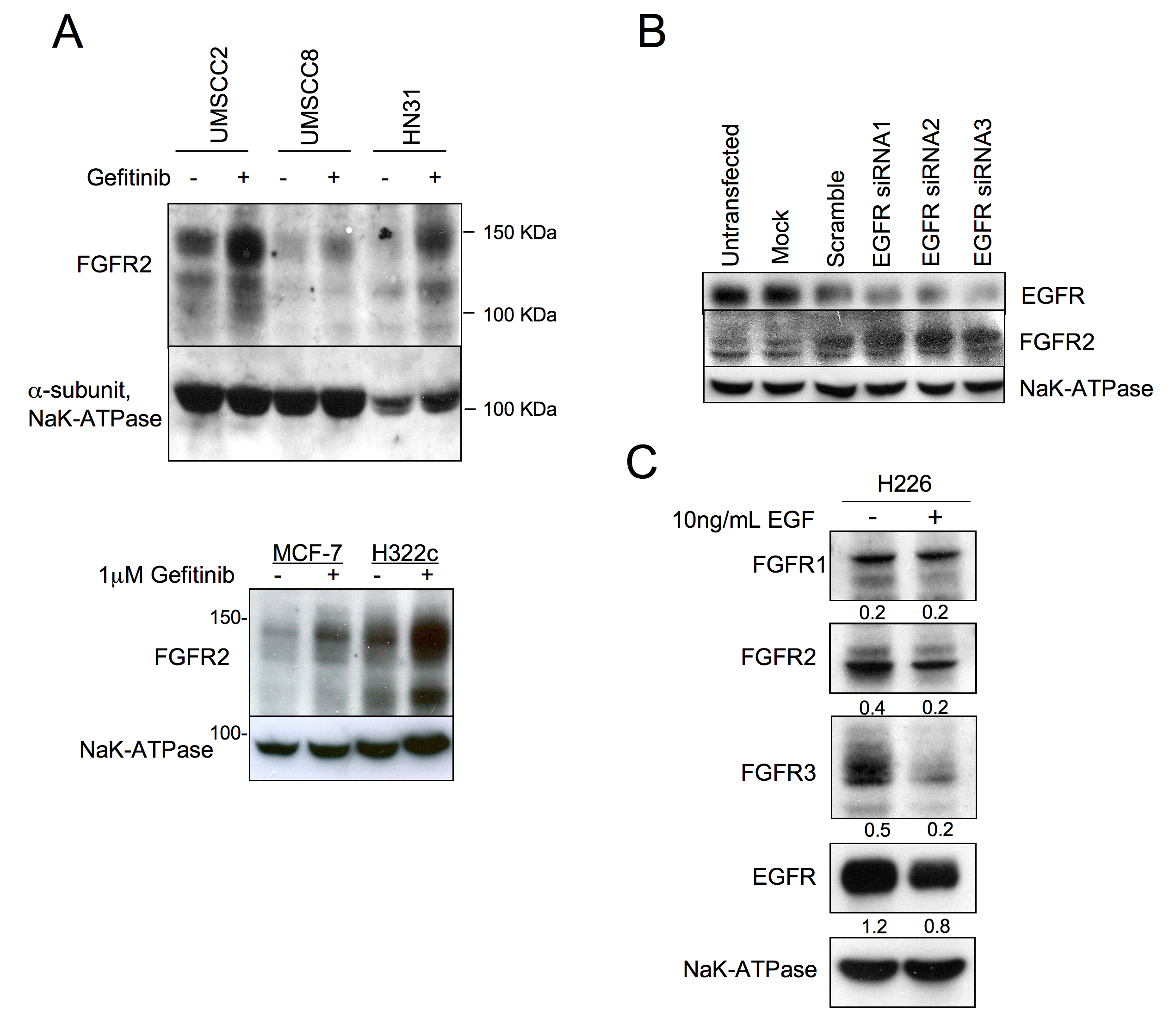

Supplement: Figure S1 — FGFR2 protein is regulated downstream EGFR signaling in cancer cell lines of epithelial origin. A. Cell lysates from the indicated HNSCC (UMSCC2, UMSCC8, HN31) and breast cancer (MCF-7) cell lines that had been treated with or without 1μM gefitinib (72hrs) were immunoblotted for FGFR2 and the α-subunit of the NaK-ATPase as a loading control. B. H322c cells transfected with 3 independent EGFR siRNA or scramble control (Sigma, St. Louis, MO) were cultured 72 hrs and immunoblotted for the EGFR, FGFR2 and NaK-ATPase. C. H226 cells were cultured with PBS or EGF (10 ng/ml) for 72 hrs. Cell lysates were immunoblotted for FGFR1, FGFR2, FGFR3, EGFR and the α-subunit of the NaK-ATPase. (1.42 MB TIF) [file pone.0014117.s001.tif]

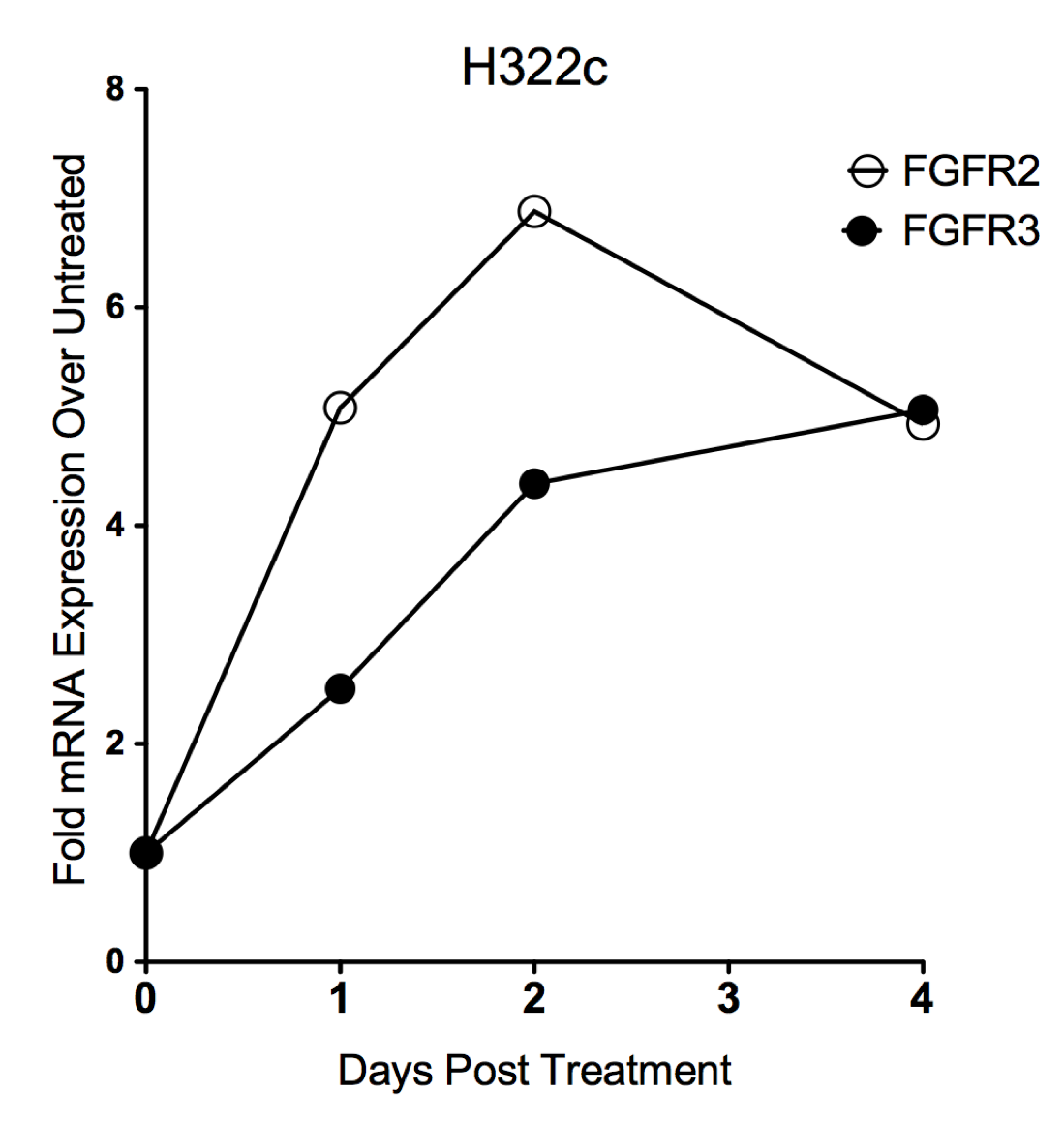

Supplement: Figure S2 — Rapid induction of FGFR2 and FGFR3 mRNA. Quantitative RT-PCR assay for FGFR2 and FGFR3 mRNAs after treatment with 1μM gefitinib for varying amounts of time was performed on total RNA from H322c cells and normalized for GAPDH mRNA levels. Data are shown as fold expression over DMSO treated cells at the indicated times. The results are a representative of 3 independent experiments. (0.18 MB TIF) [file pone.0014117.s002.tif]

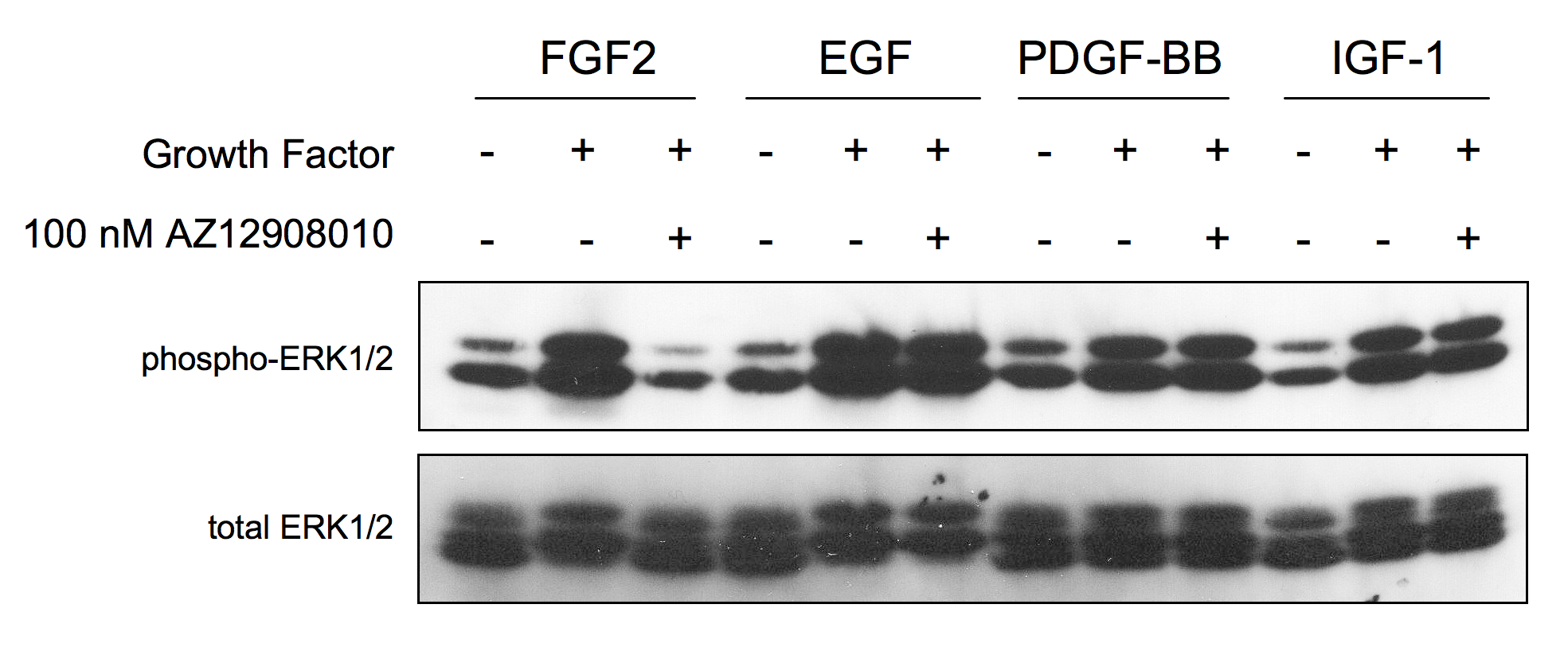

Supplement: Figure S3 — AZ12908010 is a specific inhibitor of FGFR receptors. Human gingival fibroblasts (HGF-1) purchased from ATCC were treated for 2 hrs with or without 100 nM AZ12908010 and then for another 15 minutes with or without FGF2 (10 ng/mL), EGF (10 ng/mL), PDGF-BB (20 ng/mL) or IGF-1 (10 ng/mL) as indicated. Cell extracts were prepared and submitted to SDS-PAGE and immunoblotted for phospho-ERK. The filters were subsequently stripped and reprobed for total ERK1 and ERK2 to verify equal loading. Only FGF2-stimulated phospho-ERK was inhibited by AZ12908010. (0.56 MB TIF) [file pone.0014117.s003.tif]

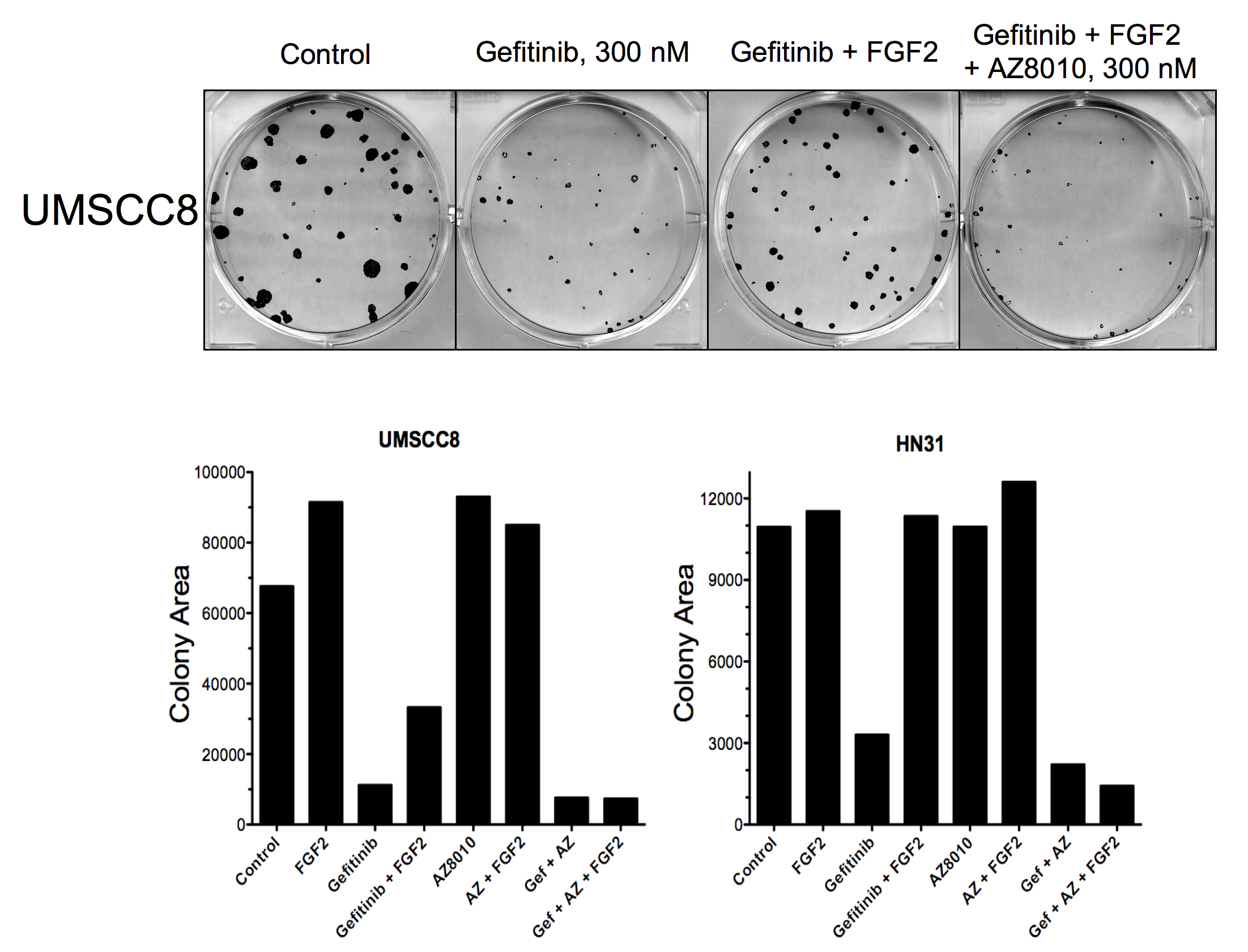

Supplement: Figure S4 — FGF2 rescues EGFR TKI dependent growth inhibition in HNSCC cells. UMSCC8 and HN31 head and neck squamous cell carcinoma lines were submitted to the clonogenic growth assay in the presence and absence of gefitinib and/or AZ12908010, an FGFR specific TKI (see Supplementary Figure S3). Colonies were stained and quantified as described in Materials and Methods. (1.26 MB TIF) [file pone.0014117.s004.tif]

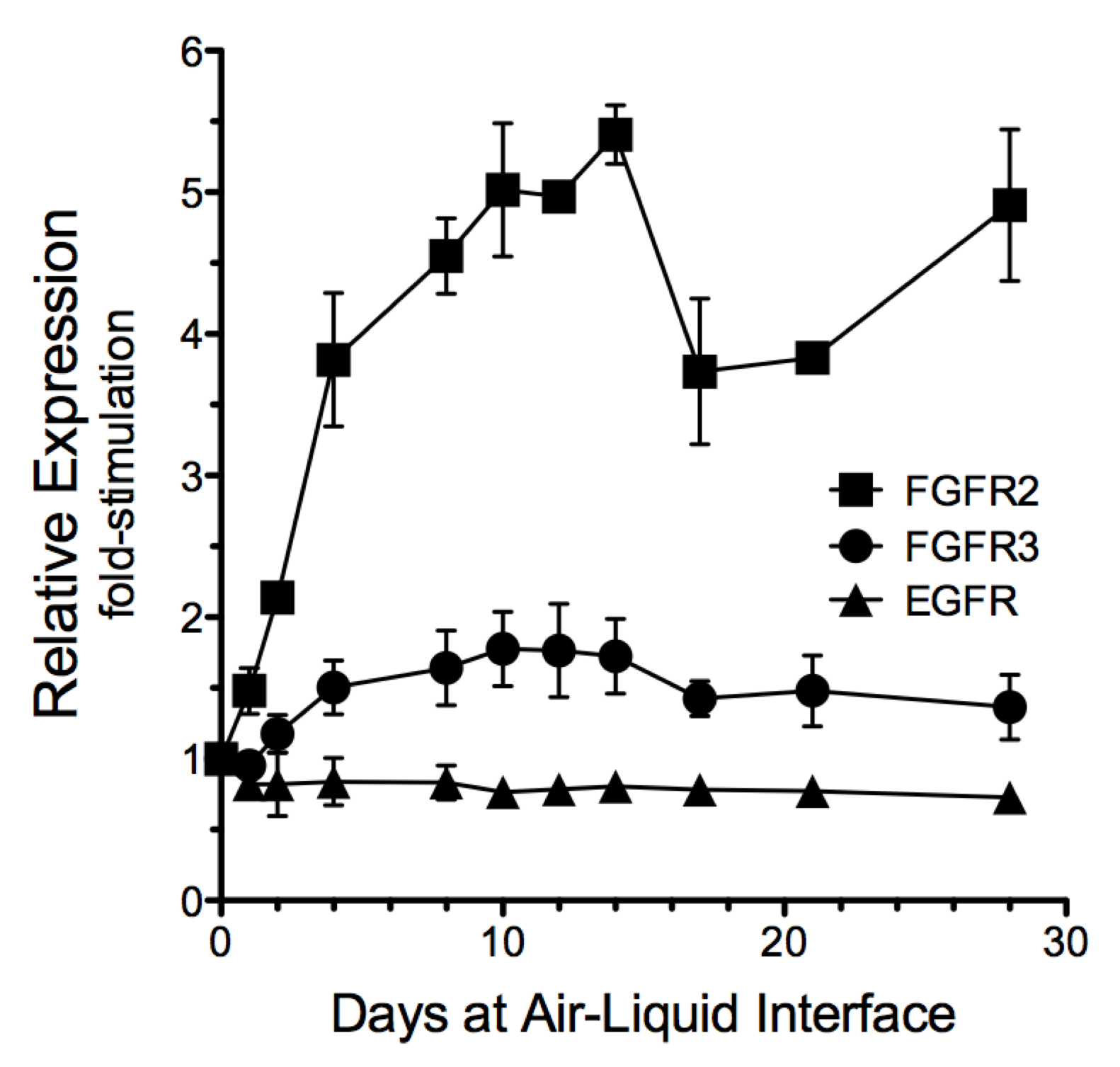

Supplement: Figure S5 — FGFR2 and FGFR3 mRNA are induced during human bronchial epithelial cell differentiation at the air-water interface. GEO Data Set GSE5264 containing Affymetrix Human Genome U133 Plus 2.0 arrays of human bronchial epithelial cells grown over a 28 day period at the air-water interface (28) were queried for expression of EGFR, FGFR2 and FGFR3 using the Affymetrix IDs EGFR (201983_s_at), FGFR2 (203638_s_at), and FGFR3 (204379_s_at). Following normalization for GAPDH expression, the data were plotted to show the relative mRNA expression of these genes over time at the air-liquid interface. The data points reflect the mean and SEM of the three independent experiments performed. (0.42 MB TIF) [file pone.0014117.s005.tif]
