# Supplementary material for: Rapidly Acquired Resistance to EGFR Tyrosine Kinase Inhibitors in NSCLC Cell Lines through De-Repression of FGFR2 and FGFR3 Expression
Source: PLoS One. 2010 Nov 29;5(11):e14117. doi: 10.1371/journal.pone.0014117 (PMC2994708; doi:10.1371/journal.pone.0014117)
Supplement: Table S1 — Gene expression changes in response to gefitinib treatment in H322c cells. Total RNA from H332c cells treated for 4 days with DMSO or 1 μM gefitinib was submitted to Affymetrix human U133 plus 2.0 arrays. Expression levels of selected tyrosine kinases and ligands are listed below. ("A" indicates absent and "P" present as assessed by the Affymetrix software program). (0.37 MB DOC) [file pone.0014117.s006.doc]

Supplementary Table 1

| **H322c** | | | | | |
| --- | --- | --- | --- | --- | --- |
| **GENE** | **AFFY ID** | **DMSO** | | **1 μM gefitinib** | |
| **FGFR1** | 207822_at | 22.3 | A | 23.4 | A |
|  | 207937_x_at | 48.1 | A | 69 | A |
|  | 210973_s_at | 88.9 | A | 184.5 | P |
|  | 211535_s_at | 207.3 | A | 306.1 | P |
|  | 215404_x_at | 61.2 | A | 88.6 | A |
|  | 222164_at | 43.1 | P | 47.6 | P |
|  | 226705_at | 119.5 | P | 156.1 | P |
|  |  |  |  |  |  |
| **FGFR2** | 203638_s_at | 81.3 | P | 2607 | P |
|  | 203639_s_at | 28 | A | 666.7 | P |
|  | 208225_at | 17.9 | A | 2.2 | A |
|  | 208228_s_at | 157.6 | P | 1724.2 | P |
|  | 208229_at | 31 | P | 27 | P |
|  | 208234_x_at | 6 | A | 6.6 | A |
|  | 211398_at | 3.7 | A | 6.1 | A |
|  | 211399_at | 5 | A | 19.7 | A |
|  | 211400_at | 2.4 | A | 1.8 | A |
|  | 211401_s_at | 30.1 | A | 264.5 | P |
|  | 240913_at | 6.5 | A | 4.5 | A |
|  |  |  |  |  |  |
| **FGFR3** | 204379_s_at | 1421.5 | P | 5057.7 | P |
|  | 204380_s_at | 43.3 | P | 29.6 | A |
|  |  |  |  |  |  |
| **FGFR4** | 204579_at | 417.3 | P | 360.1 | P |
|  | 211237_s_at | 184.1 | P | 178.8 | P |
|  | 1554961_at | 28.6 | A | 25.3 | A |
|  | 1554962_a_at | 35.4 | A | 31.3 | A |
|  |  |  |  |  |  |
| **FLT1** | 204406_at | 24.5 | A | 16.2 | A |
|  | 210287_s_at | 4.4 | A | 2.1 | A |
|  | 222033_s_at | 20.5 | A | 26.8 | A |
|  | 232809_s_at | 89.6 | A | 38.2 | M |
|  | 226497_s_at | 11.9 | A | 27.6 | P |
|  | 226498_at | 2.8 | A | 0.9 | A |
|  |  |  |  |  |  |
| **FLT4** | 210316_at | 262.4 | P | 203 | P |
|  | 229902_at | 84.1 | P | 112.6 | P |
|  | 234379_at | 4.7 | A | 4.2 | A |
|  |  |  |  |  |  |
| **KDR** | 203934_at | 2.7 | A | 21.2 | P |
|  |  |  |  |  |  |
| **IGF1R** | 203627_at | 1405 | P | 1179.2 | P |
|  | 203628_at | 414.8 | P | 426 | P |
|  | 208441_at | 16.8 | A | 12 | A |
|  | 225330_at | 1455.9 | P | 2498 | P |
|  | 243358_at | 35 | A | 35.2 | A |
|  |  |  |  |  |  |
| **MET** | 203510_at | 1158 | P | 315.1 | P |
|  | 211599_x_at | 273.7 | P | 85.4 | A |
|  | 213807_x_at | 64.6 | A | 34.7 | A |
|  | 213816_s_at | 50.8 | P | 18.8 | A |
|  |  |  |  |  |  |
| **EGFR** | 201983_s_at | 1876 | P | 5712 | P |
|  | 201984_s_at | 2856.2 | P | 4474.5 | P |
|  | 210984_x_at | 408.5 | P | 238.7 | P |
|  | 211550_at | 3.3 | A | 2.1 | A |
|  | 211551_at | 39.7 | A | 33 | P |
|  | 211607_x_at | 444.9 | P | 182.5 | P |
|  | 1565483_at | 19.6 | A | 19.7 | A |
|  | 1565484_at | 16.3 | A | 42.8 | A |
|  |  |  |  |  |  |
| **ERBB2** | 210930_s_at | 40.8 | A | 47.5 | P |
|  | 216836_s_at | 2155.2 | P | 5101.1 | P |
|  | 234354_x_at | 69.5 | P | 29.2 | P |
|  |  |  |  |  |  |
| **ERBB3** | 202454_s_at | 1823.8 | P | 3501.5 | P |
|  | 215638_at | 63.9 | P | 29.7 | A |
|  | 226213_at | 2419.7 | P | 4588 | P |
|  | 1563252_s_at | 5.3 | A | 5.8 | A |
|  | 1563253_s_at | 112.7 | A | 71.5 | A |
|  |  |  |  |  |  |
| **ERBB4** | 206794_at | 4.1 | A | 14.1 | A |
|  | 214053_at | 20 | A | 53.3 | A |
|  | 233494_at | 10 | A | 1.1 | A |
|  | 233498_at | 25 | P | 19.4 | P |
|  |  |  |  |  |  |
| **MERTK** | 206028_s_at | 458.4 | P | 917.5 | P |
|  | 211912_at | 14.1 | A | 4.9 | A |
|  | 211913_s_at | 349.4 | P | 588.2 | P |
|  |  |  |  |  |  |
| **AXL** | 202685_s_at | 65.2 | A | 82.4 | A |
|  | 202686_s_at | 360.8 | P | 1424.4 | P |
|  |  |  |  |  |  |
| **TYRO3** | 211431_s_at | 46.8 | A | 98.7 | P |
|  | 211432_s_at | 205.3 | A | 359 | P |
|  |  |  |  |  |  |
| **ALK** | 208211_s_at | 35.8 | P | 93.2 | P |
|  | 208212_s_at | 51.4 | A | 157.1 | P |
|  |  |  |  |  |  |
| **PDGFRA** | 203131_at | 2.1 | A | 16.7 | A |
|  | 211533_at | 3.8 | A | 0.7 | A |
|  | 215305_at | 19.1 | A | 14.9 | A |
|  | 1554828_at | 2.7 | A | 13.4 | A |
|  |  |  |  |  |  |
| **PDGFRB** | 202273_at | 4.1 | A | 10.1 | A |
|  |  |  |  |  |  |
|  |  |  |  |  |  |
|  |  |  |  |  |  |
| **FGF1** | 205117_at | 4.7 | A | 58.6 | A |
|  | 208240_s_at | 21.8 | A | 26.7 | A |
|  | 1552721_a_at | 9.7 | A | 15.9 | A |
|  |  |  |  |  |  |
| **FGF10** | 231762_at | 6 | A | 1.4 | A |
|  |  |  |  |  |  |
| **FGF11** | 227271_at | 200.5 | P | 393.9 | P |
|  | 231803_at | 5.6 | A | 3.5 | A |
|  |  |  |  |  |  |
| **FGF12** | 207501_s_at | 166.8 | P | 178.8 | P |
|  | 214589_at | 7.6 | A | 67.5 | P |
|  |  |  |  |  |  |
| **FGF13** | 205110_s_at | 34.8 | M | 207.8 | P |
|  |  |  |  |  |  |
| **FGF14** | 221310_at | 14.8 | A | 0.6 | A |
|  | 230231_at | 15.7 | A | 3.3 | A |
|  | 230288_at | 30.4 | A | 33.2 | A |
|  | 231523_at | 11.5 | A | 2.4 | A |
|  |  |  |  |  |  |
| **FGF16** | 221374_at | 8.7 | A | 2.7 | A |
|  |  |  |  |  |  |
| **FGF17** | 221376_at | 4.8 | A | 8.7 | A |
|  |  |  |  |  |  |
| **FGF18** | 206987_x_at | 2.7 | A | 17.9 | A |
|  | 211029_x_at | 3.2 | A | 16.8 | A |
|  | 211485_s_at | 6.5 | A | 26.9 | A |
|  | 214284_s_at | 27.8 | A | 18.6 | A |
|  | 231382_at | 25 | A | 18.5 | A |
|  |  |  |  |  |  |
| **FGF19** | 223761_at | 31.1 | A | 23.9 | A |
|  |  |  |  |  |  |
| **FGF2** | 204421_s_at | 1.5 | A | 20.7 | A |
|  | 204422_s_at | 8.6 | A | 0.5 | A |
|  |  |  |  |  |  |
| **FGF20** | 220394_at | 12 | A | 2.6 | A |
|  |  |  |  |  |  |
| **FGF21** | 221433_at | 10.1 | A | 4.7 | A |
|  |  |  |  |  |  |
| **FGF22** | 221315_s_at | 12.5 | A | 25.7 | A |
|  | 1566814_at | 8.4 | A | 8.8 | A |
|  | 1566816_at | 7.4 | A | 13.6 | A |
|  |  |  |  |  |  |
| **FGF23** | 221166_at | 8.3 | A | 2.9 | A |
|  |  |  |  |  |  |
| **FGF3** | 214571_at | 2.5 | A | 3.4 | A |
|  |  |  |  |  |  |
| **FGF4** | 206783_at | 31 | A | 24.4 | A |
|  | 1552982_a_at | 27.5 | A | 12.7 | A |
|  |  |  |  |  |  |
| **FGF5** | 208378_x_at | 10.4 | A | 1.6 | A |
|  | 210310_s_at | 1.6 | A | 1.3 | A |
|  | 210311_at | 21.2 | A | 41.1 | P |
|  |  |  |  |  |  |
| **FGF6** | 208417_at | 52.9 | A | 78.8 | A |
|  |  |  |  |  |  |
| **FGF7** | 205782_at | 11.6 | A | 20.3 | A |
|  | 1554741_s_at | 40.3 | A | 36.4 | P |
|  | 1555102_at | 12.3 | A | 0.3 | A |
|  | 1555103_s_at | 7.1 | A | 2.4 | A |
|  |  |  |  |  |  |
| **FGF8** | 208449_s_at | 1.8 | A | 4.1 | A |
|  |  |  |  |  |  |
| **FGF9** | 206404_at | 26.5 | A | 29.4 | A |
|  | 239178_at | 5.6 | A | 12.1 | A |
|  |  |  |  |  |  |
| **FGFBP1** | 205014_at | 17211.4 | P | 2257.2 | P |
|  |  |  |  |  |  |
| **FGFBP2** | 223836_at | 40.1 | A | 10.7 | A |
|  |  |  |  |  |  |
| **FGFBP3** | 238453_at | 109.2 | P | 67 | A |
|  |  |  |  |  |  |
| **AREG** | 215564_at | 4 | A | 5.5 | A |
|  | 205239_at | 1791 | P | 322.6 | P |
|  |  |  |  |  |  |
| **EREG** | 205767_at | 744.1 | P | 335.2 | P |
|  | 1569583_at | 4.3 | A | 2 | A |
|  |  |  |  |  |  |
| **HBEGF** | 203821_at | 372.8 | P | 134.8 | P |
|  | 222076_at | 6.6 | A | 2.1 | A |
|  | 38037_at | 198.6 | P | 39.2 | A |
|  | 244857_at | 4.3 | A | 13.1 | A |
|  |  |  |  |  |  |
| **EGF** | 206254_at | 89.8 | P | 68.1 | P |
|  |  |  |  |  |  |
| **TGFA** | 205015_s_at | 210.2 | P | 98.6 | A |
|  | 205016_at | 2128.7 | P | 1657.9 | P |
|  | 211258_s_at | 196.4 | P | 125.7 | M |
|  |  |  |  |  |  |
| **BTC** | 207326_at | 15.9 | A | 8.7 | A |
|  | 241412_at | 1.3 | A | 2.9 | A |
|  |  |  |  |  |  |
| **NRG1** | 206237_s_at | 36.3 | A | 114.7 | P |
|  | 206343_s_at | 649.4 | P | 382.2 | P |
|  | 208230_s_at | 21.5 | A | 62 | A |
|  | 208231_at | 28.4 | M | 2.9 | A |
|  | 208241_at | 17.7 | A | 20.9 | A |
|  |  |  |  |  |  |
| **NRG2** | 206879_s_at | 74.4 | M | 49.6 | P |
|  | 208062_s_at | 1.6 | A | 1.6 | A |
|  |  |  |  |  |  |
| **NRG3** | 229233_at | 8.9 | A | 0.5 | A |
|  |  |  |  |  |  |
| **NRG4** | 242426_at | 27.4 | P | 25.4 | A |
